# EXPLORING AND CHARACTERIZING MULTIMORBIDITY PROFILES AMONG ADULTS WITH HEART FAILURE

**DOI:** 10.1093/geroni/igae098.3010

**Published:** 2024-12-31

**Authors:** Shirin Hiatt, Paula Meek, Christopher Lee, Quin Denfeld

**Affiliations:** Oregon Health & Science University, Portland, Oregon, United States; University of Utah, Salt Lake City, Utah, United States; Boston College, Chestnut Hill, Massachusetts, United States; Oregon Health & Science University, Portland, Oregon, United States

## Abstract

Multimorbidity (MM) or coexistence of ≥2 chronic conditions is prevalent among older adults and results in poor outcomes. Among adults with heart failure (HF), MM poses exponentially more challenges, but our understanding of MM in HF is limited. The purpose of this study was to explore and characterize MM profiles in a cohort of adults with HF. We performed a secondary analysis of combined data from 6 HF studies. We used latent class analysis to identify MM profiles based on comorbidities identified with the Charlson Comorbidity Index. After identifying MM profiles, we examined sociodemographic, clinical and symptom characteristics across each profile. The sample (n=523) was 37% women, mean age 58.3±14.0 years, and mean of 2.1±1.1 comorbidities. Most patients (n=480; 92%) had MM, including HF. Among patients with MM, we identified four distinct MM profiles: Class 1-high complexity (4.6%), Class 2-moderate complexity (31.7%), Class 3-low complexity (31.9%), and Class 4-no complexity (31.9%). Class 1 was older, living alone, significantly comorbid, reported poor/fair health and depressive symptoms, and frail. Classes 2 & 3 were the next oldest but majority lived with someone. Class 4 was the youngest with the shortest duration of HF diagnosis. In conclusion, we found four distinct profiles of MM among patients with HF with marked differences in characteristics. As the prevalence of MM increases among older adults and those with chronic conditions such as HF, it is imperative to understand profiles of MM and how they contribute to function and wellbeing in order to develop effective interventions.

